# Impact of High-Hydrostatic Pressure Treatments Applied Singly or in Combination with Moderate Heat on the Microbial Load, Antimicrobial Resistance, and Bacterial Diversity of Guacamole

**DOI:** 10.3390/microorganisms8060909

**Published:** 2020-06-16

**Authors:** Javier Rodríguez López, Mª. José Grande, Rubén Pérez-Pulido, Antonio Galvez, Rosario Lucas

**Affiliations:** Microbiology Division, Department of Health Sciences, Faculty of Experimental Sciences, University of Jaén, 23071 Jaén, Spain; jrlopez@ujaen.es (J.R.L.); mjgrande@ujaen.es (M.J.G.); rppulido@ujaen.es (R.P.-P.); rlucas@ujaen.es (R.L.)

**Keywords:** guacamole, antimicrobial resistance, high pressure processing, bacterial diversity

## Abstract

Guacamole is an avocado sauce highly appreciated for its pleasant taste and nutritional value. The present study addressed the impact of high-hydrostatic pressure (HP) treatments on the product safety and bacterial diversity. Four HP treatments, 5 min each, were applied: (A) 450 megapascals (MPa) at 22 °C; (B) 450 MPa at 50 °C; (C) 600 MPa at 22 °C; (D) 600 MPa at 50 °C. Controls and treated samples were refrigerated stored for 50 days. The residual surviving fraction was lowest for the 600 MPa treatment at 50 °C. Bacterial growth on media supplemented with antibiotics (cefotaxime and imipenem) or the biocide benzalkonium chloride was detected only from control samples but not from HP-treated samples. High throughput sequencing analysis indicated that the bacterial diversity of control samples was dominated by members of Fam. Enterobacteriaceae, but it changed to a lactic acid microbiota during storage. HP-treated samples showed reduced relative abundances of Enterobacteriaceae and lactic acid bacteria and higher abundances of Pantoea, Ralstonia and Methylobacterium. Results from the study indicate that HP treatments of guacamole at 50 °C show higher microbial inactivation compared to 22 °C. However, all treatments reduced the levels of Enterobacteriaceae and penem-tolerant bacteria and provided product stability against acidification by lactic acid bacteria.

## 1. Introduction

The fruit of avocado (*Persea americana* Mill.) has a high nutritional content and is rich in vitamins, minerals, folates, potassium, and fiber, with a unique composition of lipids [1,2]. It contains several vitamins such as β-carotene, vitamin E, retinol, ascorbic acid, thiamine, riboflavin, niacin, pyridoxine, and folic acid [3,4,5]. In addition, it contains high levels of bioactive phytochemicals such as tocopherols, carotenoids, and sterols that possess antioxidant and free radical scavenging activities [6,7]. One of the most popular food products made from avocado is guacamole. Guacamole sauce contains mashed avocado pulp mixed with various condiments. In Southern Spain, guacamole production and export is an important commercial activity [8].

Fresh guacamole has been linked to foodborne disease outbreaks in the United States [9]. In order to improve the product safety and shelf life, commercial guacamole is usually processed by high-hydrostatic pressure (HP). Several studies have reported on the effects of HP treatments on the microbial load, biochemical and sensory aspects or polyphenoloxidase activity in guacamole or avocado paste [10,11,12,13]. Previous studies carried out in culture media or in other food systems have shown that the efficacy of HP treatments may increase in combination of moderate heat [14,15,16,17,18]. Yet, we have found no published report on application of HP in combination with heat on guacamole.

Improving the efficacy of HP treatments may be important in order to reduce the surviving microbial fraction that often remains in the treated samples. Surviving fractions in HP-treated samples deserve further attention not only because they could proliferate and spoil the treated product under proper conditions (like, for example, interruption of the cold chain) but also because of the risk of transmission of unwanted traits such as antimicrobial resistance through the treated product. This issue has seldom been investigated. Furthermore, the characterization of the microbial composition of the surviving fraction would also throw light on the changes in the microbial populations of guacamole occurring after application of HP treatments and the possible changes in the surviving fraction that may happen during storage. There are no previous studies based on culture-independent, next-generation sequencing technologies on the impact of HP treatments on the bacterial diversity of guacamole.

The aim of the present study was to determine the impact of different HP treatments (singly or in combination with moderate heat at 50 °C) on the microbial load, microbiological safety, and bacterial diversity of guacamole. In order to determine the impact of HP treatments on the levels of antimicrobial resistance in guacamole, we chose one representative of quaternary ammonium compounds (benzalkonium chloride) and two representatives of penem antibiotics (carbapenem and imipenem). The rationale for choosing these antimicrobials was that biocide tolerance caused by the extensive use of biocides such as quaternary ammonium compounds could be involved in coselection of antibiotic resistance [19,20] and also because of the growing concern on the dissemination of carbapenemase-producing bacteria through the food chain, not only in animal production and the environment [21,22] but also in vegetable foods [23,24]. The results of the study indicate that HP treatment remarkably changes the bacterial diversity of guacamole, with a possible additional beneficial effect related to reduction of the risks for transmission of antimicrobial resistance.

## 2. Materials and Methods

### 2.1. Preparation of Guacamole Samples

Freshly-made guacamole (made from avocado paste plus seasoning ingredients meeting the standard for Mediterranean guacamole in Southern Spain, and without any heat or pressure treatment) was provided by a local producer. Guacamole was transported on ice to the laboratory and processed the following day. Guacamole was distributed in polyethylene-polyamide bags in duplicate batches (10 g per bag) right before application of treatments.

### 2.2. High-Hydrostatic Pressure Treatments

High-hydrostatic pressure (HP) treatments were carried out by using a Stansted Fluid Power LTD HP equipment (SFP, Harlow, UK) suited with a 2.5 L vessel capable of operating in a pressure range of 0 to 700 megapacals (MPa). The system was suited with an electrical heating unit (SFP) operating in a temperature range from room temperature up to 90 °C. The following HP treatments were applied for 5 min: (A) 450 MPa at 22 °C; (B) 450 MPa at 50 °C; (C) 600 MPa at 22 °C; and (D) 600 MPa at 50 °C. Come-up speed was 75 MPa/min. Decompression was almost immediate. Pressurization fluid was water with added 10% propylenglycol (Panreac, Madrid, Spain). Control samples without HP treatments were run in parallel. Right after treatments, all samples were placed on an ice basket for 30 min and then stored at 4 °C for up to 50 days.

### 2.3. Microbiological Analysis

Immediately after application of HP treatments (time 0) and during refrigeration storage at 4 °C (days 5, 10, 20, 30, 40, and 50), two bags from controls and two from the HP-treated batches were analyzed. The content of each bag was mixed individually with 20 mL sterile saline solution and homogenized in a Stomacher 400 (Seward, Worthing, UK) for 1 min. The pH of the resulting homogenates was measured with a pH meter (Crison Instruments, S.A., Barcelona, Spain). Homogenates were serially diluted in sterile saline solution and plated in triplicate on trypticase soya agar (TSA; Scharlab, Barcelona) for total aerobic mesophiles, MacConkey agar (Scharlab) for Enterobacteriaceae and on yeast glucose agar with added chloramphenicol (YGC; Sigma Aldrich, Madrid) at 100 mg/L for yeasts and molds. The incubation period was 24 h at 37 °C (TSA, MacConkey) or 48 h at 28 °C (YGC). In parallel, samples were plated on media supplemented with antimicrobials as follows: Mueller–Hinton agar (Scharlab) supplemented with benzalkonium chloride (Sigma-Aldrich) at 200 mg/L final concentration (incubation at 30 °C under anaerobic conditions for 24 h); MacConkey agar (Scharlab) supplemented with cefotaxime (Laboratorios Normon, Madrid) at 64 mg/L or imipenem (Aurovitas, Madrid) at 4 mg/L (incubation at 37 °C for 24 h in both cases); and *Klebsiella pneumoniae* carbapenemase (KPC) agar with added supplement (Sigma) (incubation in duplicate under aerobic conditions and in anaerobic environment at 37 °C for 24 h). At the end of incubation, the numbers of colonies on the plates were counted and the data were used to calculate the viable cell counts expressed as log10 colony-forming units (CFU) per gram of sample.

### 2.4. DNA Extraction

For each sampling point and replicate, aliquots (5 mL) from each homogenate prepared as described in Section 2.3 were mixed in sterile 50 mL test tubes and centrifuged at 600× *g* for 5 min in order to remove solids. An aliquot (1.5 mL) of the resulting supernatant was then transferred to an Eppendorf test tube and centrifuged at 13.500× *g* for 5 min to recover microbial cells. The pellets were resuspended in 0.5 mL sterile saline solution each. Then, propidium monoazide (PMA™, GenIUL, S.L, Barcelona, Spain) was added to block subsequent PCR amplification of DNA from dead cells [25,26]. DNA from PMA-treated cells was extracted by using a DNeasy PowerSoil Kit (Qiagen, Madrid, Spain). The resulting DNA from the two batch replicates and same sampling point was pooled into a single sample. DNA concentration and quality were measured with a NanoDrop spectrophotometer (Thermo Scientific, Glasgow, UK). The quality and quantity of the extracted DNA was determined by QuantiFluor^®^ ONE dsDNA system (Promega, Madison, WI, USA).

### 2.5. DNA Sequencing and Analysis

The 16S rDNA V3-V4 regions were amplified following Illumina Metagenomic Sequencing Library Preparation protocol (Illumina, Inc., San Diego, CA, USA) targeting the 16S rDNA gene V3 and V4 region. Illumina adapter overhang nucleotide sequences were added to the gene-specific sequences. The following 16S rDNA gene amplicon PCR primer sequences were used: forward primer: 5′TCGTCGGCAGCGTCAGATGTGTATAAGAGACAGCCTACGGGNGGCWGCAG; and reverse primer: 5′GTCTCGTGGGCTCGGAGATGTGTATAAGAGACAGGACTACHVGGGTATCTAATCC [27]. Microbial genomic DNA (5 ng/μL in 10 mM Tris pH 8.5) was used to initiate the protocol. After 16S rDNA gene amplification, the multiplexing step was performed using Nextera XT Index Kit (Illumina). A total of 1 μL of the PCR product was run on a Bioanalyzer DNA 1000 chip to verify the size (expected size ~550 bp). After size verification, the libraries were sequenced using a 2 × 300 pb paired-end run on a MiSeq Sequencer according to manufacturer’s instructions (Illumina). Quality assessment was performed by the use of prinseq-lite program [28]. The sequence data were analyzed using qiime2 pipeline [29]. Denoising, paired-ends joining, and chimera depletion was performed starting from paired ends data using DADA2 pipeline [30]. Taxonomic affiliations were assigned using the Naive Bayesian classifier integrated in quiime2 plugins and the SILVA_release_132 database [31]. Statistical analysis was carried out with SPSS software v. 24 (IBM Corp., Foster City, CA, USA).

### 2.6. Statistical Analysis

The statistical significance of data corresponding to the culture-dependent microbiological analysis was determined by one-way ANOVA and Tukey’s test. Data on bacterial diversity were compared by Principal Coordinates Analysis (PCoA).

## 3. Results

### 3.1. Effect of Pressure Treatments on Microbial Load and Antimicrobial Resistance

Viable counts for total aerobic mesophiles (TSA) in the control samples increased gradually and significantly (*p* < 0.05) during refrigerated storage (Table 1). All the high-pressure treatments reduced viable cell counts significantly (*p* < 0.05) compared with untreated controls. Lowest reductions were obtained for samples pressurized at 22 °C (ca. 2.3 log cycles for pressures of 450 and 600 MPa), with no significant differences between both pressure treatments. Highest reductions of viable cell counts were obtained for samples pressurized at 50 °C, with statistically significant differences (*p* < 0.05) between the reductions obtained at 450 MPa (2.8 log cycles) and 600 MPa (3.30 log cycles). Compared with control samples, viable counts in the HP-treated samples only increased slightly during storage (reaching up to 3.30 to 3.94 log CFU/g for the samples pressurized at 22 °C or 2.50 to 3.39 log CFU/g for the samples pressurized at 50 °C). Furthermore, a decrease in total viable counts was detected for the highest intensity treatment by the end of storage. Samples pressurized at 600 MPa and 50 °C showed the lowest viable counts for the whole storage period compared to the rest of samples and also indicated a decrease in final counts by the end of storage.

Viable counts for presumptive Enterobacteriaceae (MacConkey agar) in the control samples did not increase during refrigerated storage of the samples, but rather decreased gradually (*p* < 0.05) and were below the detection limit (<1.48 log CFU/g) at day 40 or slightly above (1.55) at day 50 (Table 1). All high-pressure treatments reduced viable counts of presumptive Enterobacteriaceae below the detection limit for all storage times.

The populations of yeasts and molds (YGC) followed a similar trend as total aerobic mesophiles (Table 1). Counts obtained on YGC increased gradually during storage of the samples, reaching concentrations (5.87 to 6.46 log CFU/g) that were similar to counts of total aerobic mesophiles at days 20 and 30 of storage. The pressure treatments reduced viable counts for yeasts and molds in a way that depended on pressure intensity. The treatment of lowest intensity (450 MPa at 22 °C) failed to reduce viable counts below detection levels (1.48 log CFU/g), but the surviving population in the treated samples was either below detectable levels (days 5, 10, 30, and 50) or did not proliferate above 3.24 log CFU/g during storage. For treatment at 600 MPa at 22 °C, viable counts only were above detection level at day 20. For the pressure treatments applied at 50 °C, viable yeasts and molds were below the detection limit at all sampling points.

The pH of control samples decreased gradually and significantly (*p* < 0.05) from 4.53 at time 0 to 4.07 at day 50 (Table 1). None of the pressure treatments induced a significant decrease in the pH of samples (*p* > 0.05 at T0). Furthermore, the pH of the pressurized samples did not decrease significantly during storage (with the exception of treatment A at day 40). At day 50, all treated samples showed similar pH values comprised between 4.34 and 4.35.

In order to determine the load of carbapenem-tolerant microorganisms, sample homogenates and serial dilutions were plated on media containing carbapenem and imipenem. Viable counts obtained on MacConkey agar supplemented with carbapenem were low but still detectable at days 20, 30, and 40 (Table 2). Growth on MacConkey agar supplemented with imipenem was only detected at days 10, 20, and 30. Viable counts on KPC agar with added supplement incubated under aerobic conditions increased remarkably for days 20 to 30 (resembling the viable count increase detected on TSA) and still remained high at day 50. Colonies were green-bluish and white. By contrast, growth on KPC agar incubated under anaerobic conditions was only detected for days 0 to 10 (pink colonies only), and the counts were very low (Table 2). In order to test biocide tolerance, samples were also plated on Mueller–Hinton agar supplemented with benzalkonium chloride. However, growth was only obtained from control homogenates at day 0, and the counts were very low (Table 2).

### 3.2. Bacterial Diversity

The bacterial diversity recovered from guacamole, pressurized or not and chill stored was determined by Illumina sequencing of the V3–V4 variable regions of the 16S rRNA gene. The numbers of assigned reads and alpha diversity indexes of controls and pressurized samples are shown in Table 3. The lowest Shannon and Simpson diversity indices were observed in samples treated by HP.

**Figure 1:**
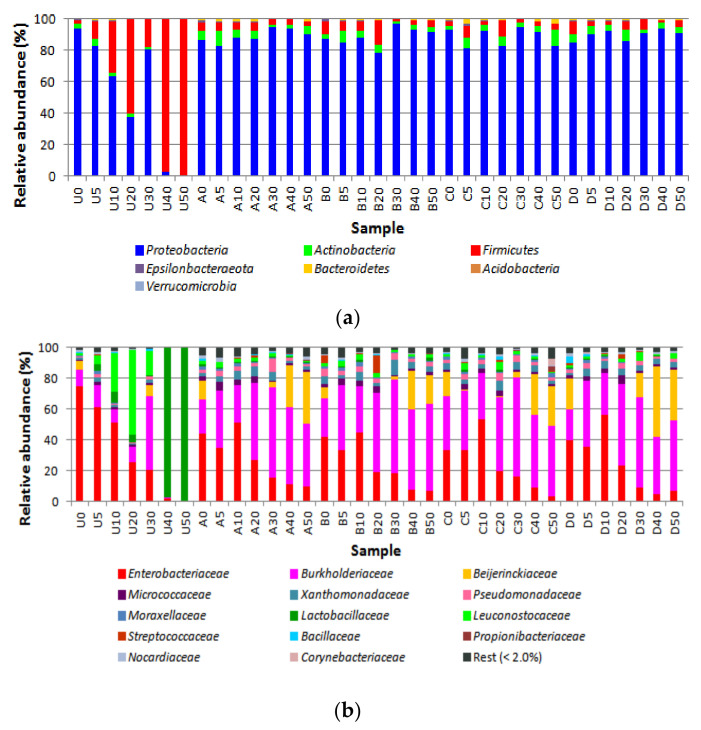

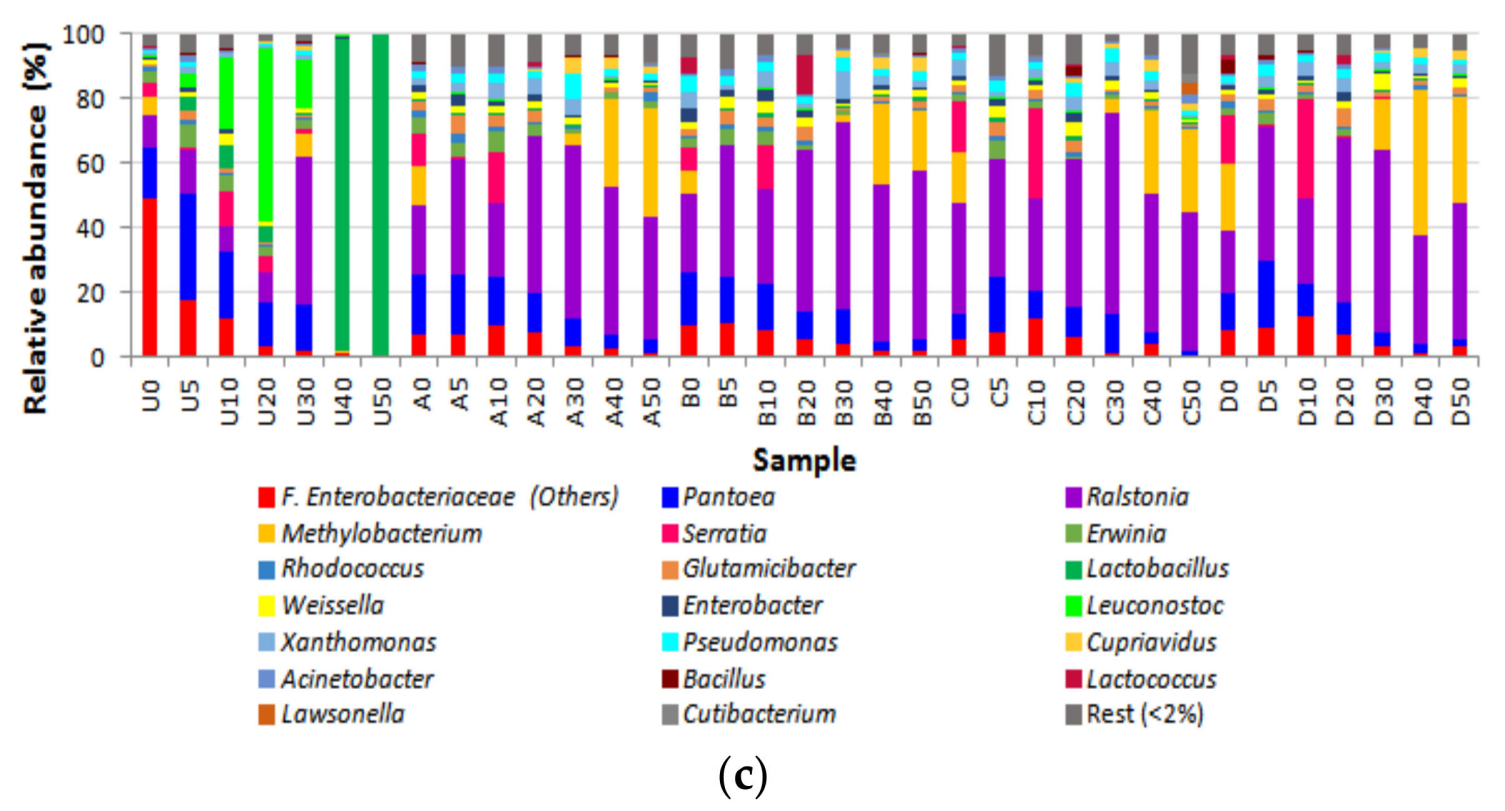
Bacterial diversity of guacamole samples at phylum (**a**), family (**b**), and genus (**c**) levels. U, untreated controls. HP treatments: A, 450 MPa at 22 °C; B, 450 MPa at 50 °C; C, 600 MPa at 22 °C; and D, 600 MPa at 50 °C. Numbers indicate incubation time (days).

The different operational taxonomic units (OTUs) found in guacamole samples were grouped into 6 phyla (Figure 1A), of which Proteobacteria were by far the main representatives, followed by Firmicutes and Actinobacteria. These phyla included representatives of 49 families (plus 2 higher taxons). Of them, 14 families with relative abundances of at least 2% covered between 93 and 99.9% of OTUs (Figure 1B). At the genus level, a total of 98 genera (plus 6 higher taxons) were detected. Of them, 20 had relative abundances of at least 2% and covered between 87.2 and 99.9% of OTUs (Figure 1C).

Fam. Enterobacteriaceae was the main bacterial group found in the control samples at time 0. At the genus level, OUTs assigned to Enterobacteriaceae (others) were predominant, followed by OTUs assigned to genus Pantoea. During storage, there was a marked decrease in the relative abundance of Fam. Enterobacteriaceae and in particular OTUs assigned to Enterobacteriaceae (others) while OTUs assigned to genus Pantoea increased at days 5 and 10. Major changes detected in the relative abundances of other families included an increase of Fam. Leuconostocaceae (gen. Leuconostoc) at days 10 and 20, followed by Fam. Burkholderiaceae (gen. Ralstonia) at day 30 and Fam. Lactobacillaceae (gen. Lactobacillus) at days 40 and 50.

In the HP treated samples, there was a general reduction in the relative abundance of Fam. Enterobacteriaceae both after treatment and during storage. Within this family, samples from HP treatments were characterized by a general reduction in the relative abundance of Enterobacteriaceae (others) and an early increase in the relative abundance of OTUs assigned to gen. Pantoea. The relative abundance of Burkholderiaceae (represented by gen. Ralstonia) increased both after treatment and during storage. Ralstonia became the predominant group in most of the samples during storage, while Pantoea decreased during storage. Methylobacterium (Fam. Beijerinckiaceae) became a relevant OTU by the end of storage (days 40 and 50) in all treated samples, regardless of treatment. Methylobacterium showed highest relative abundances (45.3% and 32.6%) in samples from treatment D at days 40 and 50. Members of families Bacillaceae and Lactobacillaceae had very low relative abundances in the HP-treated samples. Fam. Leuconostocaceae was represented in the treated samples by genus Weissella instead of Leuconostoc. Weissella was detected in most of the treated during early-to-mid storage, at relative abundances up to 5.3%.

**Figure 2:**
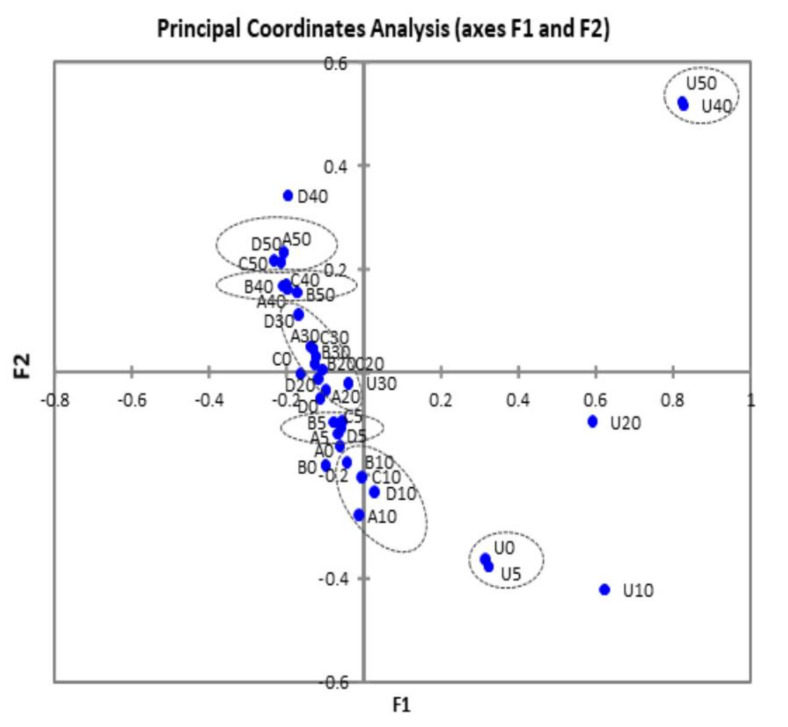
Principal coordinates analysis of guacamole samples treated or not by HP. U, untreated controls. HP treatments: A, 450 MPa at 22 °C; B, 450 MPa at 50 °C; C, 600 MPa at 22 °C; and D, 600 MPa at 50 °C.

Principal Coordinates Analysis (PCoA) revealed that most of the control samples mapped separately from the treated samples (Figure 2). Furthermore, control samples U20, U40, and U50 did not show significant positive correlations (*p* > 0.05) with any of the treated samples. It could also be observed that the treated samples mapped in subgroups according to storage time rather than treatment, as exemplified by the subgroups 1 (samples A5-B5-C-5-D5), 2 (samples A10-B10-C10-D10), 3 (all treated samples from days 20 and 30), 4 (samples A40-B40-C40), and 5 (samples A50-C50-D50).

## 4. Discussion

Results obtained in the present study indicated that high-pressure (HP) treatments failed to completely inactivate microbial populations in guacamole. Previous studies also reported the presence of a residual fraction of viable cells in vegetable foods and beverages after high-pressure treatments [32,33]. One study [12] reported a surviving fraction of ca. 2 log units per gram (total aerobic mesophiles and lactic acid bacteria) in avocado paste treated at 600 MPa (3 min) and refrigerated stored for 45 days.

The presence of a residual surviving fraction has been attributed to the tailing effects observed when vegetative cells are treated by HP [34]. It has been reported that tailings tend to disappear when HP is combined with heat [35]. The results from the present study indicated that treatments applied at 50 °C achieved significantly higher reductions of viable counts (total aerobic mesophiles) in guacamole compared to treatments applied at 22 °C and reduced viable counts of yeasts and molds below detection levels for all storage times. These results could be explained by the greater inactivation effect of the combined treatment on vegetative cells rather than on spore forms. According to previous studies, HP at 50 °C may not be sufficient to inactivate bacterial endospores [18]. However, being a high-acid food (pH < 4.6), it seems unlikely that endospore germination and extensive bacterial growth would occur in the treated guacamole under refrigeration. It has also been reported that the spores of heat resistant molds may require the combination of high pressure and thermal processing for their inactivation in high-acid foods, while the less resistant mold spores can be inactivated by HP at room temperature [18]. In addition, yeast ascospores were reported to have lower resistances than bacterial and mold spores and were much easier to inactivate by HP [18]. Most of these results are based on studies carried out on fruit juices and juice concentrates, where the sugar content may have an effect on spore inactivation. The results from the present study suggest that the combined treatments of HP at 50 °C improved the inactivation of yeasts and molds, since no viable counts were obtained in any of the treated samples compared to samples pressurized at 22 °C.

A second issue addressed in the present study was the impact of HP treatments on the load of antimicrobial resistance in guacamole. First, we chose benzalkonium chloride (BC) for biocide tolerance as a representative of quaternary ammonium compounds (QACs) because QACs are widely used for cleaning and disinfection in the food industry and also because biocide tolerance could be involved in coselection of antibiotic resistance [19,20]. However, we only detected bacterial growth from control samples at time 0, but not during storage or after application of HP treatments. Second, we focused on carbapenem tolerance because there is a growing concern on the dissemination of carbapenemase-producing bacteria through the food chain [21,22,23,24]. The results obtained indicated low levels of presumptive carbapenem-tolerant (cefotaxime, imipenem) enterobacteria in guacamole, but also indicated that the tolerant bacteria could persist in the sauce during refrigerated storage for up to 30–40 days. More worrying, viable counts obtained on KPC agar incubated under aerobic conditions increased dramatically during refrigerated storage of the control samples. Although KPC agar was designed for the detection of carbapenem-tolerant Enterobacteriaceae from clinical samples, carbapenemase-producing strains of *Pseudomonas aeruginosa* and *Acinetobacter baumannii* as well as other intrinsically tolerant bacteria could also grow in this medium (which lacks the selectivity of MacConkey agar). That would explain the much lower counts obtained when KPC agar was incubated under anaerobiosis. In order to obtain more information on the genetic background of penem tolerance, a collection of bacterial strains isolated from the selective media has been obtained and is currently under investigation.

There is a risk that ingestion of food containing penem-tolerant bacteria may result in transfer of the genetic determinants of resistance to intestinal microbiota. However, we should remark that no bacterial growth was detected on media supplemented with antibiotics in any of the pressurized guacamole samples. Thus, a low intensity treatment of 450 MPa at 22 °C for 5 min would improve the safety of guacamole by reducing the load of penem-tolerant bacteria below detectable levels.

Analysis of the dynamics of bacterial populations during refrigerated storage of untreated guacamole samples indicated a shift in the microbiota from members of Enterobacteriaceae and other Proteobacteria to a lactic acid fermentation. This change was followed by a decrease in pH during storage and a succession of lactic acid bacteria. The population of Leuconostoc increased in relative abundance from days 5 to 20, and Lactobacillus was the predominant OTU in the samples towards the end of storage. This succession is typical of some vegetable fermentations such as sauerkraut fermentation [36]. Leuconostoc are less tolerant to low pH than lactobacilli. At the same time, lactobacilli have a higher capacity for acidification and utilization of residual sugars and are expected to take over in the last stages of the fermentation and create a selective environment that results in displacement of other bacterial populations.

Application of HP treatments not only had an impact on the microbial load of guacamole samples but also affected its bacterial diversity, reducing the relative abundances of Enterobacteriaceae and increasing that of Ralstonia. The pressurized samples also showed some changes in bacterial diversity during storage, but these changes differed markedly from control samples. First, lactic acid bacteria (LAB) had very low relative abundances in the pressurized samples during storage. Lactobacillus was below 1.5%, and Leuconostoc was below 0.3%. Weissella was the only LAB detected in the treated samples, especially during early-to-mid storage times. It is tempting to suggest that Weissella displaced Leuconostoc in the HP-treated samples, possibly because of a higher tolerance to the HP treatments or a higher capacity to thrive in the pressurized samples. *Weissella* spp. have been isolated from a wide range of habitats, e.g., plants, vegetables, and fermented foods such as European sourdoughs and Asian and African traditional fermented foods [37]. The growth capacity of Weissella in the refrigerated guacamole could be limited by the low temperature. However, some Weissella species (e.g., *W. diestrammenae* and *W. soli*, *W. viridescens*) have been reported to grow in a temperature range from 4 to 37 °C [37].

Nowadays, it is important for the food industry not only to evaluate the relationships between food processing treatments and the product shelf life but also to understand the impact of treatments on the food safety. Results from the present study indicate that all the HP treatments tested reduce the levels of Enterobacteriaceae and carbapenem-resistant bacteria and provide product stability against acidification by lactic acid bacteria. Application of HP treatments at 50 °C achieved additional reductions of viable cell counts and reduced the concentrations of survivors during storage. Therefore, HP treatments at 50 °C could be recommended in cases of risk of higher contamination (like for example, when using raw materials with high microbial loads). Further studies are needed in order to evaluate the incidence of antimicrobial resistance in guacamole.

## Figures and Tables

**Table 1:** Viable cell counts and pH of guacamole samples treated or not by HP.

Total Aerobic Mesophiles	T0	T5	T10	T20	T30	T40	T50
Controls	4.89 ± 0.09	5.03 ± 0.11	5.98 ± 0.13 ^a^	5.79 ± 0.21 ^a^	6.70 ± 0.12 ^a^	6.55 ± 0.06 ^a^	6.25 ± 0.19 ^a^
Treatment A	2.57 ± 0.14 ^b^	3.29 ± 0.10 ^b^	3.24 ± 0.14 ^b^	3.30 ± 0.18 ^b^	3.27 ± 0.15 ^b^	3.21 ± 0.27 ^b^	3.24 ± 0.13 ^b^
Treatment B	2.07 ± 0.12 ^b,c^	2.70 ± 0.14 ^b,c^	2.85 ± 0.17 ^b^	2.51 ± 0.17 ^b,c^	2.59 ± 0.09 ^b,c^	3.39 ± 0.13 ^b^	2.43 ± 0.10 ^b,c^
Treatment C	2.60 ± 0.11 ^b^	3.34 ± 0.16 ^b^	3.32 ± 0.14 ^b^	3.40 ± 0.08 ^b^	3.42 ± 0.14 ^b^	3.94 ± 0.20 ^b^	3.14 ± 0.21 ^b^
Treatment D	1.55 ± 0.15 ^b,d^	2.08 ± 0.15 ^b,d^	2.50 ± 0.16 ^b,d^	2.12 ± 0.13 ^b,d^	2.42 ± 0.11 ^b,d^	1.82 ± 0.26 ^b,d^	1.75 ± 0.20 ^b,d^
**Enterobacteriaceae**	**T0**	**T5**	**T10**	**T20**	**T30**	**T40**	**T50**
Controls	4.63 ± 0.14	4.48 ± 0.12	4.17 ± 0.14 ^e^	3.61 ± 0.13 ^e^	2.55 ± 0.12 ^e^	<1.48	1.55 ± 0.15 ^e^
Treatment A	<1.48	<1.48	<1.48	<1.48	<1.48	<1.48	<1.48
Treatment B	<1.48	<1.48	<1.48	<1.48	<1.48	<1.48	<1.48
Treatment C	<1.48	<1.48	<1.48	<1.48	<1.48	<1.48	<1.48
Treatment D	<1.48	<1.48	<1.48	<1.48	<1.48	<1.48	<1.48
**Yeasts and molds**	**T0**	**T5**	**T10**	**T20**	**T30**	**T40**	**T50**
Controls	2.55 ± 0.12	2.83 ± 0.27	4.79 ± 0.10 ^a^	5.87 ± 0.18 ^a^	6.46 ± 0.16 ^a^	5.60 ± 0.29 ^a^	5.33 ± 0.20 ^a^
Treatment A	1.82 ± 0.26 ^f^	<1.48	<1.48	2.17 ± 0.22 ^f^	<1.48	3.24 ± 0.24 ^f^	<1.48
Treatment B	<1.48	<1.48	<1.48	<1.48	<1.48	<1.48	<1.48
Treatment C	<1.48	<1.48	<1.48	1.60 ± 0.09	<1.48	<1.48	<1.48
Treatment D	<1.48	<1.48	<1.48	<1.48	<1.48	<1.48	<1.48
**pH**	**T0**	**T5**	**T10**	**T20**	**T30**	**T40**	**T50**
Controls	4.53 ± 0.01	4.34 ± 0.02 ^g^	4.35 ± 0.03 ^g^	4.33 ± 0.01 ^g^	4.30 ± 0.01 ^g^	4.23 ± 0.01 ^g^	4.07 ± 0.08 ^g^
Treatment A	4.47 ± 0.01	4.41 ± 0.01	4.44 ± 0.03	4.41 ± 0.01	4.35 ± 0.08	4.25 ± 0.10 ^h^	4.35 ± 0.01
Treatment B	4.41 ± 0.01	4.43 ± 0.01	4.45 ± 0.01	4.43 ± 0.01	4.43 ± 0.03	4.34 ± 0.03	4.35 ± 0.03
Treatment C	4.46 ± 0.01	4.42 ± 0.03	4.44 ± 0.01	4.42 ± 0.06	4.42 ± 0.03	4.33 ± 0.04	4.34 ± 0.01
Treatment D	4.42 ± 0.04	4.45 ± 0.01	4.43 ± 0.01	4.45 ± 0.01	4.45 ± 0.03	4.36 ± 0.06	4.35 ± 0.04

HP treatments: A, 450 MPa at 22 °C; B, 450 MPa at 50 °C; C, 600 MPa at 22 °C; and D, 600 MPa at 50 °C. T, incubation time (days). The detection limit was calculated taking into consideration the dilution factor of the guacamole in the homogenate. Statistical significance (*p* < 0.05): ^a^, significantly higher than counts obtained at time 0; ^b^, significantly lower than untreated controls (all sampling points); ^c^, significantly lower than treatment A at the same storage time; ^d^, significantly lower than treatment C at the same storage time; ^e^, significantly lower than counts obtained at time 0 (Enterobacteriaceae); ^f^, significantly lower than controls (yeasts and molds) at the same storage time; ^g^, pH significantly lower compared to time 0; and ^h^, pH significantly lower compared to samples A0 to A 20.

**Table 2:** Viable cell counts of untreated guacamole samples on media containing antimicrobials.

Antimicrobial	T0	T5	T10	T20	T30	T40	T50
Cefotaxime	<1.48	<1.48	<1.48	2.85 ± 0.07	2.84 ± 0.16	1.50 ± 0.03 ^a^	<1.48
Imipenem	<1.48	<1.48	1.87 ± 0.12	3.13 ± 0.25 ^b^	1.63 ± 0.21	<1.48	<1.48
KPC aerobiosis	3.26 ± 0.12	4.13 ± 0.07 ^c^	4.63 ± 0.23 ^c^	5.64 ± 0.12 ^c^	6.17 ± 0.13 ^c^	6.05 ± 0.18 ^c^	5.49 ± 0.13 ^c^
KPC anaerobiosis	2.52 ± 0.05 ^d^	1.79 ± 0.10 ^d^	2.06 ± 0.15 ^d^	<1.48	<1.48	<1.48	<1.48
Benzalkonium chloride	2.55 ± 0.05	<1.48	<1.48	<1.48	<1.48	<1.48	<1.48

KPC, Klebsiella pneumoniae carbapenemase agar with added supplement. T, incubation time (days). No viable cells were detected on media supplemented with antimicrobials for any of the HP-treated samples. Statistical significance (*p* < 0.05): ^a^, significantly lower than other cefotaxime counts; ^b^, significantly higher than other imipenem counts; ^c^, significantly higher than KPC aerobiosis counts at time 0; and ^d^, significantly lower than counts obtained for KPC aerobiosis.

**Table 3:** No. of reads and alpha diversity indexes at genus level of guacamole samples treated or not by high-hydrostatic pressure (HP).

Sample	No Reads	Chao1	Shannon	Simpson
U0	3478	35	0.67	0.36
U5	3440	37	0.62	0.34
U10	9275	48	0.81	0.35
U20	7689	34	0.87	0.39
U30	4641	33	0.43	0.17
U40	69,978	19	0.87	0.47
U50	162,425	23	0.51	0.26
A0	2926	38	0.49	0.24
A5	2987	44	0.38	0.16
A10	2483	36	0.52	0.26
A20	2823	40	0.4	0.17
A30	2384	28	0.36	0.16
A40	4474	42	0.5	0.2
A50	3294	44	0.54	0.24
B0	2113	36	0.43	0.21
B5	4865	55	0.4	0.15
B10	1876	31	0.42	0.2
B20	2571	39	0.38	0.15
B30	2312	30	0.38	0.17
B40	3750	39	0.41	0.16
B50	4096	45	0.4	0.15
C0	2948	33	0.53	0.29
C5	1689	41	0.41	0.19
C10	6809	51	0.47	0.19
C20	1565	36	0.38	0.17
C30	1968	24	0.29	0.13
C40	2074	36	0.44	0.21
C50	8033	62	0.51	0.2
D0	7764	52	0.61	0.24
D5	4082	43	0.41	0.16
D10	4209	43	0.49	0.2
D20	3967	41	0.39	0.15
D30	5623	41	0.43	0.17
D40	4514	35	0.45	0.18
D50	6665	39	0.48	0.19

U, untreated controls. HP treatments: A, 450 MPa at 22 °C; B, 450 MPa at 50 °C; C, 600 MPa at 22 °C; and D, 600 MPa at 50 °C. Numbers indicate incubation time (days). Chao 1 is an abundance-based estimator of species richness. Shannon and Simpson indices are estimators of both species richness and species evenness, with more emphasis on either richness (Shannon) or evenness (Simpson).
